# Aluminum Doped Titania as a Support of Copper Catalysts for SCR of Nitrogen Oxides

**DOI:** 10.3390/ma14206021

**Published:** 2021-10-13

**Authors:** Wojciech Guziewicz, Anna Białas, Bogna D. Napruszewska, Małgorzata Zimowska, Jacek Gurgul

**Affiliations:** 1 Faculty of Energy and Fuels, AGH University of Science and Technology, Mickiewicza 30, 30059 Kraków, Poland; wguziewicz@student.agh.edu.pl; 2 Jerzy Haber Institute of Catalysis and Surface Chemistry, Polish Academy of Sciences, Niezapominajek 8, 30239 Kraków, Poland; ncnaprus@cyf-kr.edu.pl (B.D.N.); malgorzata.zimowska@ikifp.edu.pl (M.Z.); jacek.gurgul@ikifp.edu.pl (J.G.)

**Keywords:** sol-gel method, titanium oxide, aluminum, anatase, SCR, nitrogen oxides

## Abstract

Aluminum doped titania samples were synthesized as supports of copper oxide catalysts for NO reduction with ammonia. Samples were prepared by the sol-gel method with various ratios of aluminum to titanium. Their thermal stability was examined by TG/DSC methods which revealed that precursors were decomposed at 450 °C. The XRD measurements showed that aluminum caused the diminishing of titania crystallites and was built into the anatase structure or formed an amorphous phase. The admixture of aluminum in titania resulted in a significant increase in specific surface area of mesoporous supports as determined by low temperature sorption of nitrogen. Results of the catalytic tests over copper/aluminum-titania samples obtained by impregnation pointed out that the addition of aluminum broadened the temperature window of high catalytic activity. The increase in Al concentration shifted the temperature of maximum activity to higher values, and at the same time lowered nitrous oxide formation as well. Better catalytic efficiency could result from high copper dispersion on the catalysts surface, as well as the synergistic interaction between Ti and Cu causing reduction in CuO species as confirmed by XPS measurements. It was shown that copper was present as Cu^+^ species mainly, forming Cu-O-Ti bonds on the catalysts surface.

## 1. Introduction

Among the catalysts used in selective catalytic reduction (SCR) of nitrogen oxides (NO_x_) with ammonia, copper oxide is regarded as a highly efficient active phase. However, it must occur as isolated or dimeric species in the catalyst because crystalline CuO exhibits the narrow temperature window of its high catalytic activity [1,2]. Variable valence of copper, good dispersion, and strong acidic properties of a catalyst are crucial in SCR and were observed in coprecipitated Cu-Ti systems [1]. In such systems, tuning Lewis–Brønsted acidity, responsible for the low or high temperature range of the reaction, respectively, ensures a broad temperature window. Well-chosen support can accelerate the Cu^+^/Cu^2+^ redox cycle and decrease the amount of oxidized NH_3_, as well as can improve the adsorption ability. Thus, the main challenge in preparing copper oxide catalysts is to widen the temperature window of their high activity [1]. It can be attained by elaborating the materials which enable good dispersion of copper species on the support and which are cheaper than zeolites. Among catalyst supports for the SCR process titania and alumina are widely used [3]. Alumina increases acidic properties making a catalyst less prone to SO_2_ adsorption, thus more resistant to SO_2_ poisoning. In case of titania, a similar behavior is also reported [1]. It is worth noting that alumina supported copper catalysts are active at higher temperatures and less nitrous oxide is formed than over titania ones [4]. This is a huge advantage because N_2_O is a greenhouse gas which damages the ozone layer [4]. The N_2_O formation is determined by the reaction temperature and the content of active phase. In case of copper catalysts, the concentration of Cu reduced form is responsible for this by-product [4]. The presence of Al_2_O_3_ develops the surface of catalyst and improves its hydrothermal resistance and inertness [1]. On the other hand, the active sites are more accessible on TiO_2_ than on Al_2_O_3_ [4]. Good textural and chemical properties of supports are attained when sol-gel method is used for their synthesis [5].

The aim of the presented paper was to elaborate the synthesis of aluminum doped titania by the sol-gel method to obtain the support ensuring good copper dispersion on the catalysts for SCR of NO. The physicochemical characterization of catalysts was carried out in detail to explain the catalyst activity in relation to the aluminum concentration in the support.

## 2. Materials and Methods

Glacial acetic acid (J.T. Baker, Deventer, Holland) was added to titanium isopropoxide (Acros Organics, Geel, Belgium) in the molar ratio of 10:1 and stirred for 30 min. Then, the solution of a proper amount of aluminum nitrate (V) nonahydrate (Eurochem BGD, Tarnów, Poland) with distilled water (acid/water molar ratio equal 1:10) was added dropwise into the mixture for 40 min. The samples with Al to Ti ratio equal to 1:9, 2:8, 3:7 or 4:6, as well as the sample without aluminum were prepared in this way. Initially, some white precipitate was formed which slowly dissolved forming clear solution. Then, the solution was maintained at 80 °C for 100 min under stirring, slowly transforming into a gel. The gel was dried at 80 °C for 4 days. Obtained samples were ground and calcined at 450 °C for 3 h. This temperature was determined on the base of thermogravimetric analyses (Figure S1, Supporting Information). The supports were marked as Ti, Ti_0.9_Al_0.1_, Ti_0.8_Al_0.2_, Ti_0.7_Al_0.3_, and Ti_0.6_Al_0.4_.

The copper active phase was deposited with the incipient wetness impregnation method. Strictly defined amount of copper (II) nitrate (V) trihydrate (Acros Organics, Geel, Belgium) was dissolved in distilled water and deposited on obtained supports, resulting in 1 wt. % of copper. The precursors were dried at 80 °C overnight and calcined at 450 °C for 3 h. The catalysts were denoted as Cu/Ti, Cu/Ti_0.9_Al_0.1_, Cu/Ti_0.8_Al_0.2_, Cu/Ti_0.7_Al_0.3_, and Cu/Ti_0.6_Al_0.4_.

Thermogravimetric and differential scanning calorimetric analyses were conducted to determine the decomposition temperatures of support precursors. These analyses were carried out with a STA 409 PC Luxx by Netzsch–Gerätebau GmbH (Selb, Germany). The 20 mg samples were subjected to the thermal treatment in the temperature range of 30 °C to 1000 °C with a temperature increase rate of 10 °C min^−1^ under air flow of 30 cm^3^ min^−1^. To measure the real content of metals, the XRF method was used. By means of an EDX 3600H spectrometer by Skyray Instrument (Dallas, Texas, USA) with a tungsten lamp calibration curves for Al_2_O_3_, TiO_2_, and CuO were prepared. The experimental error for these compounds was 2.7, 7.0, and 0.2%, respectively. For light elements the lamp voltage was 9 kV, current 150 mA, and the time of a measurement was equal to 200 s, for elements heavier than Ca these parameters were 40 kV, 450 mA, and 100 s, respectively. The crystallographic structure was examined by X-ray diffraction with a PANalytical Empyrean diffractometer (Malvern, UK) with CuKα radiation (λ = 1.54056 Å). XRD patterns were recorded in the 2θ angle range of 3° to 90° with a step of 0.013°. The morphology of the solids was carried out by means of JEOL JSM—7500F field emission scanning electron microscope equipped with an retractable backscattered-electron detector (RBEI) and energy dispersive spectra (EDS) detection system of characteristic X-ray radiation INCA PentaFetx3 EDS system (Jeol Ltd., Tokyo, Japan). The low temperature sorption of nitrogen performed using a Nova 2200e sorptomat by Quantachrome Instruments (Boynton Beach, FL, USA) allowed to record isotherms and to determine the specific surface area of samples, as well as the pore size distribution and pore volume. Samples of 0.1 g were outgassed at 200 °C for 12 h. The surface area was determined by the BET method, the volume pore distribution by the BJH method and the pore volume by the single point method.

The X-ray photoelectron spectroscopy (XPS) experiments were carried out in a multi-chamber system equipped with a hemispherical analyzer (SES R4000, Gammadata Scienta, Uppsala, Sweden). The unmonochromatized Al K_α_ (1486.6 eV) X-ray source was used to generate core excitations. The anode was operating at 12 kV and 15 mA current emission. The energy resolution of the system determined for Ag 3d_5/2_ excitation line was 0.9 eV (pass energy 100 eV). The spectrometer was calibrated according to ISO 15472:2001. The base pressure in the analysis chamber was about 1 × 10^−10^ mbar and about 5 × 10^−9^ mbar during the experiment. The powder samples were examined after pressing into indium foil and mounting on a special holder. The area of sample analysis was about 4 mm^2^ (5 mm × 0.8 mm). High-resolution spectra were collected at pass energy of 100 eV (with 25 meV step), whereas survey scans at pass energy of 200 eV (with 0.25 eV step). The experimental curves were fitted in CasaXPS 2.3.23 software (Casa Software Ltd., Teignmouth, UK) with a combination of Gaussian and Lorentzian lines of variable proportions (70:30) after subtraction of the Shirley-type background. The relative intensity ratio of 2p_3/2_ and 2p_1/2_ lines in doublets was fixed to 2:1. All binding energy values were charge-corrected to the carbon C 1s excitation which set at 285.0 eV.

Selective catalytic reduction in nitrogen oxide with ammonia was performed over the obtained catalysts. N_2_O concentration was measured as well. An AO2020 IR detector by ABB Group (Frankfurt am Main, Germany) was used as an analyzer. Then, 0.2 g of the catalyst fraction between 0.25 and 1 mm, was placed in a quartz reactor and outgassed at 450 °C for 1 h in a helium flow. Then, it was cooled down to 150 °C and the tests were conducted. The concentration of NO and NH_3_ was 800 ppm, the amount of O_2_ was 3.5%, and helium was added to achieve the total flow of 100 cm^3^ min^−1^. The catalytic activity was measured within the temperature range of 150 °C to 450 °C, with a step of 50 °C attained in 5 min and maintained for 30 min. NO conversion (X_NO_) was calculated from the following equation:

(1) $$X_{\mathrm{NO}}=\frac{n_{\mathrm{NO},0}-n_{\mathrm{NO}}}{n_{\mathrm{NO},0}}\cdot 100\%$$

where: n_NO,0_ and n_NO_ are the inlet and outlet streams of NO, respectively.

## 3. Results and Discussion

### 3.1. Composition, Crystallographic Structure, and Morphology

The copper content and aluminum to titanium ratio are presented in Table 1. Some differences between intended and measured values are observed, they could result from experimental errors during syntheses or XRF measurements.

**Figure 1:**
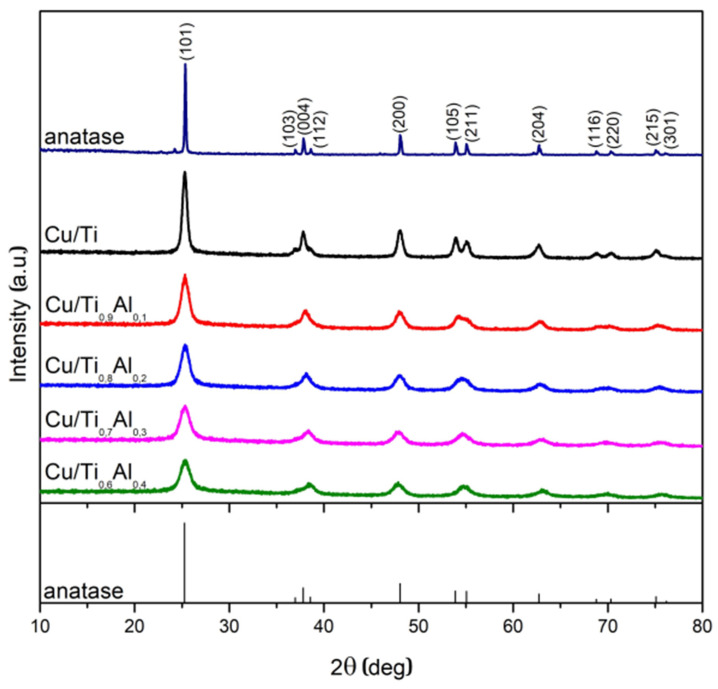
XRD patterns of Cu/Ti_x_Al_1-x_ catalysts and the reference—commercial TiO_2_ anatase compared with data [01-071-1167].

Powder XRD patterns of the Cu/Ti_x_Al_1-x_ samples are shown in Figure 1. The diffraction lines observed for the Cu/Ti sample at 2θ = 25.3°, 37.0°, 37.7°, 38.7°, 48.0°, 53.8°, 55.0°, 62.6°, 68.6°, 70.2°, 74.9°, 82.5°, can be ascribed to the (101), (103), (004), (112), (200), (105), (211), (204), (116), (220), (215), and (224) planes of the anatase structure, respectively [01-071-1167]. The addition of aluminum to the titania precursor and the copper deposition did not cause the appearance of new phases. It suggests that aluminum could build into the anatase structure or occurs as amorphous phase. One can note, that the incorporation of Al into the titania structure should be visible by a little change of the (101) peak position due to difference in the ionic radii of Al^3+^ (0.53 Å) and Ti^4+^ (0.60 Å). Because the shift of (101) peak position is not seen, we believe that introduced aluminum occurs as amorphous alumina phase. There are no traces of any copper phases in the XRD patterns which suggests that copper, present in a small concentration, occurs as a well dispersed phase.

With an increase in the aluminum content one can observe the decrease in maxima intensities, as well as widening of the peak FWHM, resulting in smaller crystallite sizes (D), which was confirmed by their estimation using the Scherrer equation:

(2) $$D=\frac{K\cdot \lambda }{\beta \cdot \cos\theta }$$

where: *K* is the shape factor (0.9), *λ* is the wavelength of X-ray (1.54056 Å), *β* is FWMH of (101) reflex at 25.3°, *θ* is the Bragg angle.

Table 1 shows that the average crystallite size of the copper catalyst supported on titania was close to 15 nm and decreases significantly after aluminum addition.

**Figure 2:**
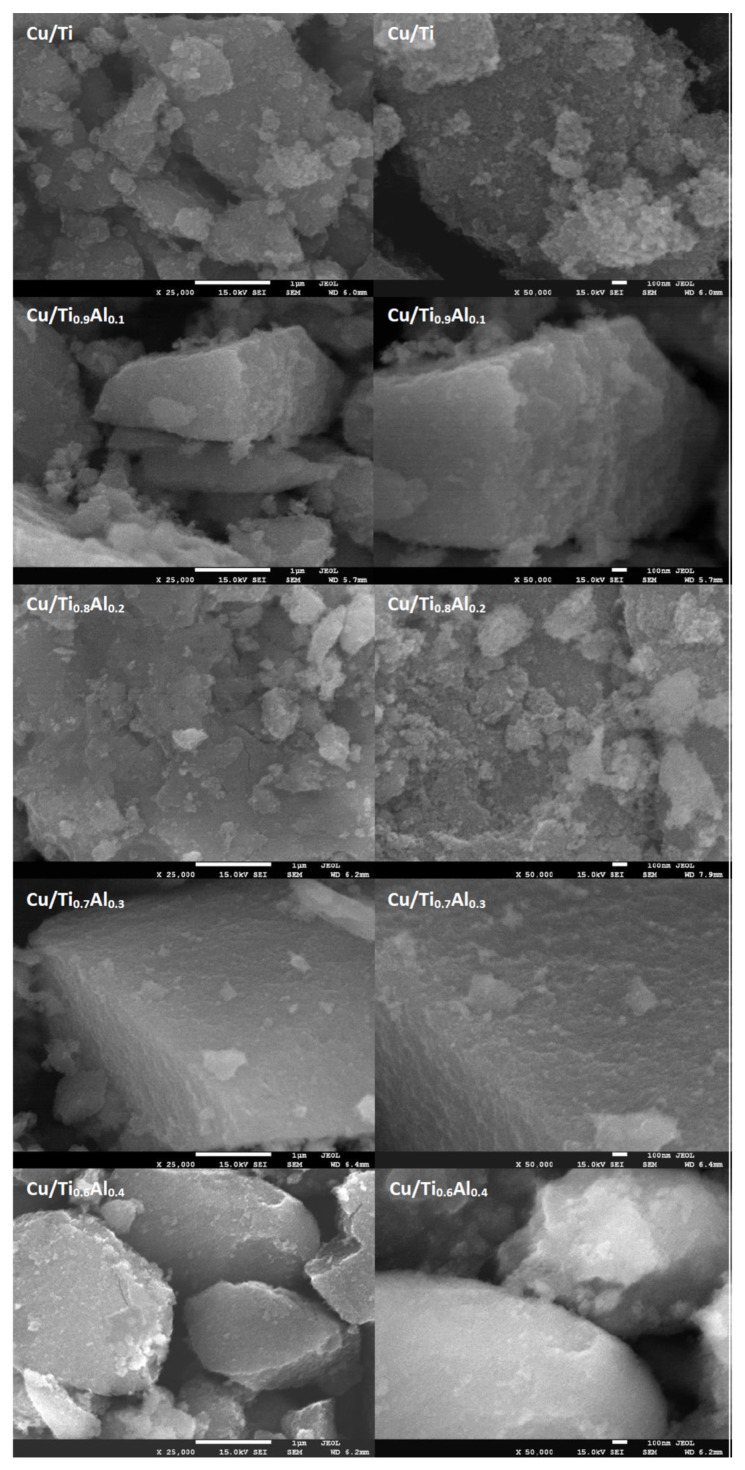
SEM images with the morphology of the Al-titania supported copper catalysts.

**Figure 3:**
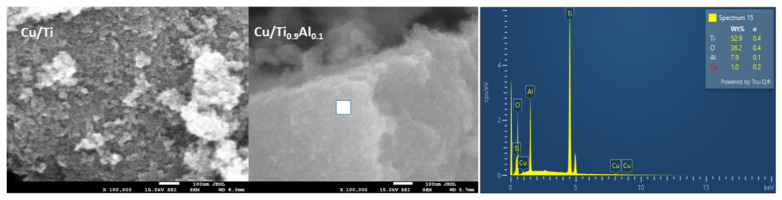
SEM images of Cu/Ti and Cu/Ti_0.9_Al_0.1_ catalysts obtained with maximum magnification. The EDX analysis performed in the selected area of Cu/Ti_0.9_Al_0.1_ sample (marked by white square) is also shown.

Figure 2 and Figure 3 show the SEM images with the morphology of the Al-titania supported copper catalysts. The titanium catalyst has a porous morphology and is characterized by the presence of tiny particles about 20–30 nm in size. The incorporation of Al into titania support results in formation of more dense materials. Comparison of SEM and XRD analyses shows that the addition of Al into titanium isopropoxide can affect the termination of the crystallites of anatase growth and, finally, sticks to TiO_2_ particles as an amorphous phase. EDX analysis (Figure 3) revealed that the amount of introduced copper varied in the range of 0.9 to 1.4 wt.% for all samples.

### 3.2. Textural Properties

**Figure 4:**
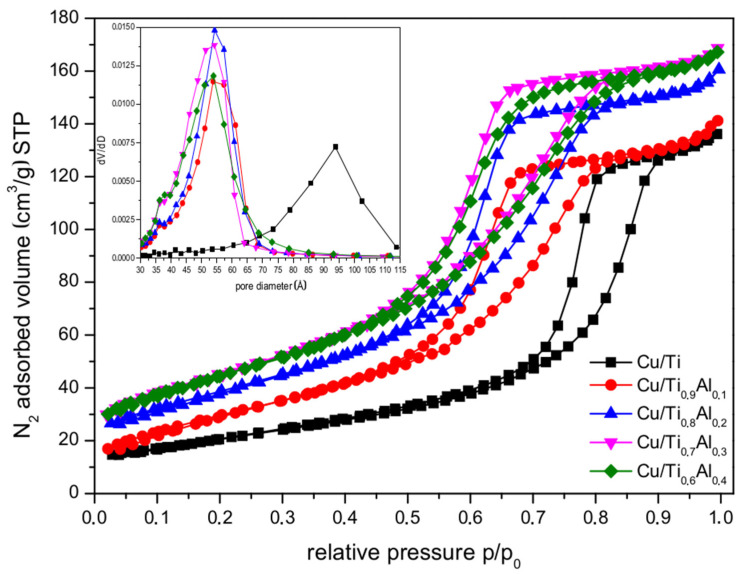
Nitrogen sorption isotherms and BJH pore distribution (inset) of Cu/Ti_x_Al_1-x_ catalysts.

In Figure 4 the nitrogen sorption isotherms of catalysts are shown. The isotherms have characteristic hysteresis loops, which classified them as Type IV according to IUPAC. These characteristic shapes of isotherms are associated with the capillary condensation taking place in larger mesopores (>4 nm). Thus, the obtained materials can be classified as mesoporous. It can be noticed that with the increased amount of aluminum, the hysteresis loops move to lower values of relative pressure suggesting reduction in the pore size. This observation was confirmed by the pore size distribution presented in the inset of Figure 4. For the titania supported catalyst the dominating pore size was 94 Å, and was attributed to the pore system predominantly form by inter-particle contacts. Aluminum doped catalysts show pore size distribution in the range of ca. 54 Å and the lowering intensity of the maximum of the peaks with increasing amount of Al in the samples. These results confirm the covering of the TiO_2_ particles with alumina containing amorphous phase and shrinking of the inter-particle spaces. An increase in the amount of Al in the support increases the quantity of the amorphous component and results in the increase in the specific surface area of the supports. It is worth noting that in Type IV materials there is a correlation between the shape of the obtained hysteresis loop and the textural properties of the adsorbent. Here, we found the difference in hysteresis shapes between Ti-Al supported catalysts (Type H2) and titania one (close to Type H1), which leads to the conclusion that the pore structure in studied materials changed as well. As seen from Table 1, the titania support exhibited BET specific surface area of 97 m^2^ g^−1^. The substitution of titanium by low amount of aluminum caused significant increase in surface area. Further, an increase in aluminum content (up to 40%) resulted in further increase in SSA_BET_, as seen for the Ti_0.7_Al_0.3_ sample, and only slight changes in the case of the Ti_0.6_Al_0.4_ one. The total pore volume increased by about 20% for all supports containing aluminum. The deposition of copper onto supports brought about the sample SSA_BET_ lowering by ca. 15–25%. As far as the total pore volume is regarded the copper addition resulted in ca. 10% decrease in all samples with the exception of the Cu/Ti_0.9_Al_0.1_ catalyst in which the pore volume is lowered by ca. 25%. In the case of this sample, the copper phase is placed mainly on the outer surface of the supporting particles.

### 3.3. Surface Composition—XPS

The relative abundance of elements at catalysts surface obtained from the XPS survey scans in depth of max. 8.5 nm are presented in Table 2. It is computed by the assumption that samples are made of pure and uniform TiO_2_ with density equal to 4.26 g cm^−3^ [6]. The calculations present 95% of all photoelectrons escaping from the surface. The high-resolution spectra of Cu 2p, Ti 2p, O 1s, C 1s, and Al 2p were used to investigate the chemical states of the active phase in the fresh and used catalysts.

**Figure 5:**
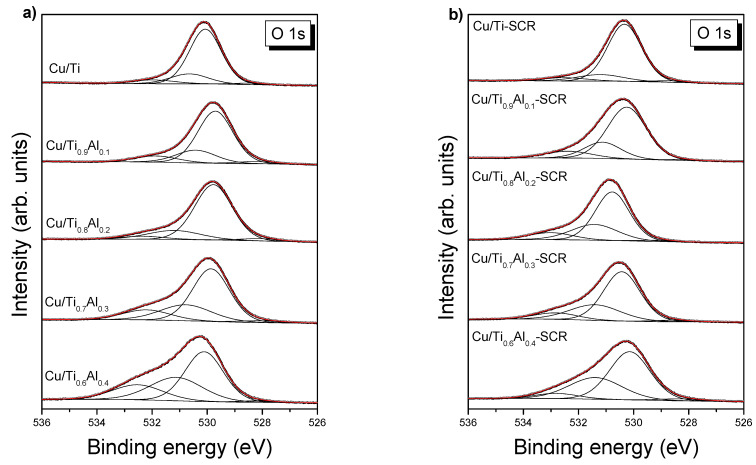
O 1s XPS spectra of fresh (**a**) and used (**b**) catalysts.

The O 1s spectra (Figure 5a) of fresh catalysts show four components: (i) a main line (over 70% of total spectrum area) located at 529.7–530.1 eV related to oxygen in titania [7], (ii) oxygen from defective CuO or Cu_2_O (BE = 530.4–531.2 eV) [8], (iii) peak at BE > 531.9 eV assigned to OH groups, and oxygen of organic contaminants, and (iv) very weak contribution at 528.0–528.4 eV, which can be referred to Cu-Ti-O bonds (Table SA1 in Supporting Information). Relative increase in aluminum in catalysts causes increase in the component with BE = 530.4–531.2 eV at the cost of reducing the area of titania component. Thus, it strongly suggests that Al-O bonds can contribute to this component. Similar BE range of O 1s line (530.5 to 531.1 eV) was referred to the thin films of Al_2_O_3_ [9]. However, Severino et al. [10] have reported O 1s component with BE in the region of 529.7 to 531.9 eV for CuAl_2_O_4_ spinel, and this possibility cannot be excluded.

The O 1s spectra of spent catalysts (Figure 5b) can be described by similar four components as well. Binding energies of oxygen components coming from titania and defective copper oxides are slightly shifted towards higher energies (Tables SA1 and SA2 in Supporting Information). It can be caused by reoxidation of samples after SCR process due to their storage under air atmosphere.

The C 1s core lines (Figure S2, Supporting Information) for fresh and used catalysts are composed of three peaks at 285.0 eV (organic contaminants), 286.0–286.6 eV (C-O groups), and 289.0–289.7 eV (O-C=O groups). The used catalysts show a bit higher content of C-O groups (20–35%) in comparison to fresh ones (9–26%), whereas the amount of O-C=O groups remains unchanged after SCR process (Supporting Information, Tables SB1 and SB2). The hydrocarbon contamination was used as an internal calibration for our samples, as we mentioned above.

Single Al 2p component was detected with Al 2p_3/2_ BE values close to 74.0 eV and 74.3 eV for fresh and used catalysts, respectively (Table 3 and Table 4). Such contribution can be associated with the Al^3+^, similar to the case of Al_2_O_3_ [11,12]. The spin-orbit splitting of Al 2p doublet was constrained to Δ_SO_ = 0.41 eV.

The Ti 2p spectra can also be well fitted by a single symmetric doublet with the spin-orbit splitting of 5.74 eV. Ti 2p_3/2_ BE values of 458.5–459.1 eV are characteristic for the presence of Ti(IV) [7,13,14,15,16]. One can identify that the highest Ti 2p_3/2_ BE of 459.4 eV was found in Cu/Ti_0.8_Al_0.2_-SCR.

**Figure 6:**
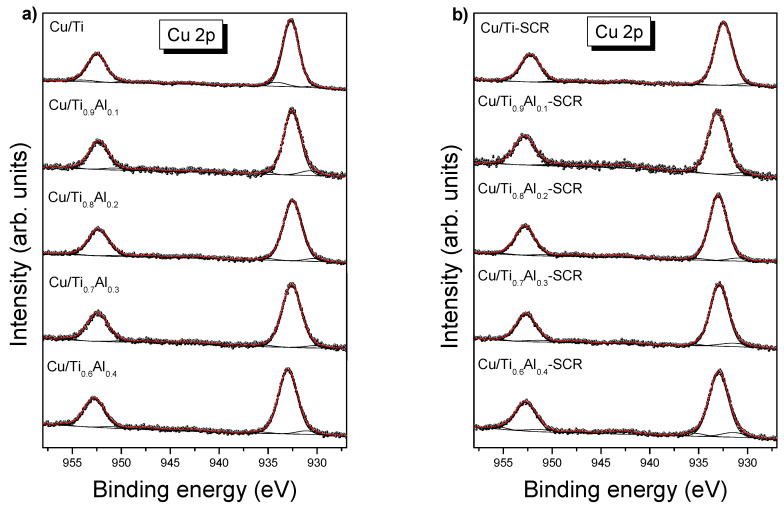
Cu 2p XPS spectra of fresh (**a**) and used (**b**) catalysts.

Figure 6 presents XPS spectra of fresh and used catalysts at Cu 2p region. In addition to well separated spin-orbit peaks with total momentum j = 3/2 and j = ½, there are much weaker additional structures between them related to shake-up satellites. The satellite peaks are characteristic of Cu(II) which has a d^9^ configuration in the ground state and may occur when the outgoing photoelectrons interacts with valence electrons exciting to higher-energy levels. The shake-up satellites are absent in d^10^ Cu^0^ or Cu^+^ spectra. It is generally accepted that much stronger shake-up structures are associated with more Cu^2+^ species in the sample [17,18].

The Cu 2p XPS spectra (Figure 6) of all catalysts are described by three components, which can be assigned to lower valence Cu (Cu^0^ at BE of Cu 2p_3/2_ < 931.4 eV and Cu^+^ at 932.5–933.0 eV) and to Cu^2+^ species (~935.0 eV). The most dominating component (>90% of total spectrum area) attributed to Cu ions in Cu-O-Ti sites with a binding energy typical of Cu^+^ demonstrates that redox reduction occurs between Cu^2+^ precursor and Ti^3+^ in surface defective sites of TiO_2_ to produce the Cu^+^ species strongly interacting with TiO_2_, as reported previously [19]. We tentatively assigned the Cu^2+^ XPS signal to CuAl_2_O_4_ or Cu(OH)_2_ for which very near BE were reported [8,11,17]. On the other hand, Shen et al. [18] reported peak at 934.9 eV as the ionic Cu^2+^ binding in CuO phase, whereas the covalent part of Cu^2+^ was attributed to 933.4 eV peak position. The distinction between copper oxidation states needs some comment. The absolute BE values of Cu 2p peaks are rarely helpful in identifying the copper oxidation state, since only small differences between Cu^+^ and Cu^0^, and between octahedral Cu^2+^ and Cu^+^ are reported in the literature [8,20,21]. In such a case, distinguishing between Cu^+^ and Cu^0^ species can be drawn from the Cu 2p_3/2_—Cu L_3_M_45_M_45_ Wagner plot [8,22]. The kinetic energy of maximum of Cu L_3_M_45_M_45_ Auger peak was obtained from the survey spectra, whereas BE of the major component from the Cu 2p high-resolution spectra (Table 5). The copper Auger parameter for such a contribution is close to 1849 eV for all measured samples. It is worth noting that Auger parameters of 1851.2 eV (metallic Cu), 1849.2 eV (Cu_2_O), 1851.3 eV (CuO) and 1850.9 eV (Cu(OH)_2_) were reported by Biesinger et al. [8,17]. Taking into account above values and drawing the copper Wagner plot, we can attribute the most intense component to Cu^+^ species [22]. This result, together with very weak shake-up satellites can point out that the dominating oxidation state of copper in all catalysts is +1.

The SCR process resulted only in a small increase in Cu^0^ contribution, especially well visible in high-Al catalysts (Table 5 and Table 6). One can see also a small BE shift of Cu^2+^ component towards higher energies. As we mentioned above, it can be caused by reoxidation of samples after SCR process due to their storage under air atmosphere.

The absence of Cu^0^/Cu^+^ XRD signals together with XPS results suggest the high dispersion of copper species on the catalyst surfaces. The synergistic interaction between Ti and Cu, which promotes the reducibility of CuO, giving rise to the formation of surface Cu^+^, as shown by XPS analysis, appears to play a significant role in determining the catalyst efficiency.

### 3.4. Catalytic Activity

**Figure 7:**
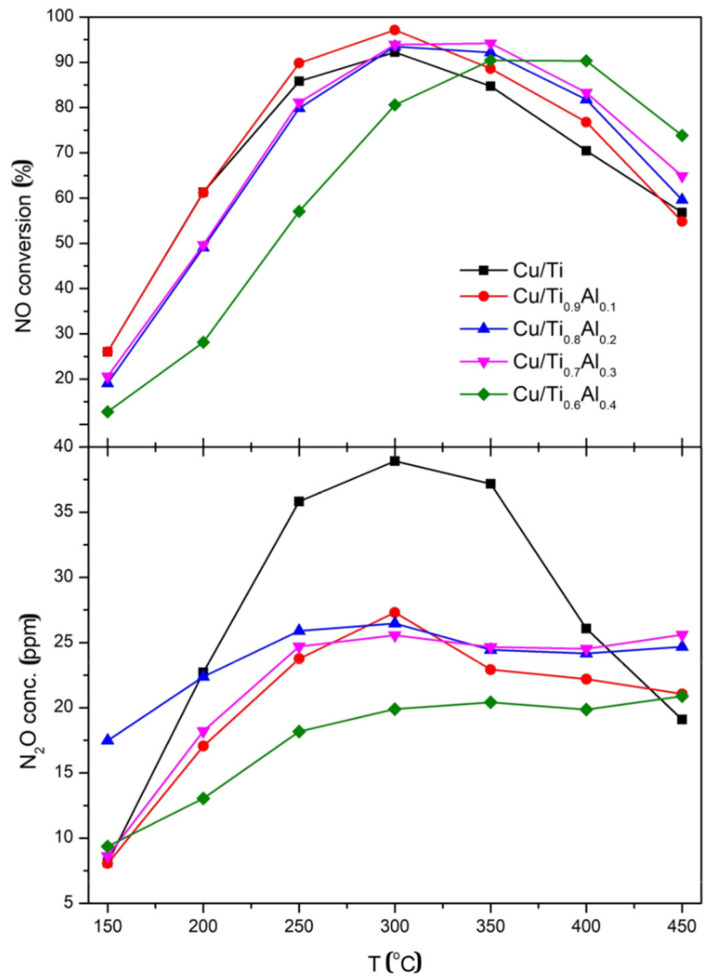
NO conversion and N_2_O concentration over Cu/Ti_x_Al_1-x_ catalysts.

NO conversion and N_2_O formation in SCR of NO with ammonia over Cu/Ti_x_Al_1-x_ catalysts are presented in Figure 7. For the Cu/Ti catalyst, with pure TiO_2_ as a support, above 80% of conversion was observed in the temperature range of 250 °C to 350 °C, with the maximum of ca. 90% at 300 °C. The substitution of 10% of titanium with aluminum resulted in a few percent increase in the catalytic activity between 250 °C and 400 °C. A further increase in Al content caused broadening of the temperature window of 80% NO conversion up to 400 °C as registered for the Cu/Ti_0.8_Al_0.2_ and Cu/Ti_0.7_Al_0.3_ samples. This better catalytic activity could result from higher copper dispersion on the catalysts surface and from its presence in Cu-O-Ti bonds in the form of Cu^+^ cations (Table 5 and Table 6). For the sample with maximum aluminum concentration Cu/Ti_0.6_Al_0.4_ the maximum activity is significantly shifted to higher temperatures, NO conversion >80% was attained in the temperature range of 300 °C to 400 °C and maximum at 400 °C. This shift could be caused by higher Al content causing the increase in Brønsted acidity. During catalytic tests the small amounts of N_2_O formation was observed. The N_2_O concentration was below the threshold limit of 30 ppm for all aluminum-doped catalysts. The lowest values were measured for the sample with the highest Al content. For the titania supported catalyst the N_2_O concentration achieved ca. 40 ppm at temperatures related to its highest catalytic activity (250–350 °C).

## 4. Conclusions

Aluminum doped titania precursors were obtained by the sol-gel method. They attained mass stability at 450 °C and after calcination and copper deposition, they were tested as catalysts in SCR of NO. XRD results showed that titania crystallized as anatase phase whereas aluminum and copper oxides remained amorphous. Specific surface area of mesoporous samples increased with aluminum concentration to above 100 m^2^ g^−1^, whereas their average pore size shifted to lower values. Catalytic tests showed that Al admixture to the titania support caused that the temperature range of high activity was broadened and shifted to higher temperatures in the case of sample with maximum aluminum content. The samples containing aluminum caused the formation of only trace amount of N_2_O. The improvement of catalytic efficiency was caused by good copper dispersion on the catalyst surface resulting from the specific surface development of aluminum doped titania supports. The copper surface species contained mainly Cu^+^ cations and occurred in Cu-O-Ti bonds. Thus, one can say that the catalyst efficiency improvement was achieved by the synergistic interaction between Ti and Cu, and hence the reducibility of CuO.

## Figures and Tables

**Table 1:** Composition, average crystallite size, and textural properties of Al-titania supported copper catalysts.

Sample		Average Crystallite Size [nm]	Surface Area * SSA_BET_ [m^2^ g^−1^]	Pore Volume * [cm^3^ g^−1^]
Nominal Formula	Measured Formula			
Cu/Ti	1.1Cu/Ti	14.6	75 (97)	0.203 (0.225)
Cu/Ti_0.9_Al_0.1_	1.3Cu/Ti_0.90_Al_0.10_	9.7	110 (129)	0.204 (0.277)
Cu/Ti_0.8_Al_0.2_	1.3Cu/Ti_0.85_Al_0.15_	8.8	131 (178)	0.233 (0.256)
Cu/Ti_0.7_Al_0.3_	1.5Cu/Ti_0.74_Al_0.26_	7.9	148 (190)	0.247 (0.274)
Cu/Ti_0.6_Al_0.4_	1.6Cu/Ti_0.63_Al_0.37_	7.5	145 (190)	0.246 (0.269)

* the values obtained for supports are shown in brackets.

**Table 2:** Surface elemental composition of fresh and used catalysts (at.%).

Sample	C	O	Al	Ti	Cu	Cl *
Cu/Ti	5.84	65.93	---	26.84	0.99	0.41
Cu/Ti-SCR	8.65	65.57	---	24.9	0.8	---
Cu/Ti_0.9_Al_0.1_	4.59	64.89	6.44	23.08	0.59	0.4
Cu/Ti_0.9_Al_0.1_-SCR	5.09	65.47	5.83	22.86	0.75	---
Cu/Ti_0.8_Al_0.2_	4.62	65.63	8.22	20.6	0.52	0.4
Cu/Ti_0.8_Al_0.2_-SCR	5.55	65.28	8.55	20.04	0.58	---
Cu/Ti_0.7_Al_0.3_	5.69	63.52	12.49	17.22	0.46	0.61
Cu/Ti_0.7_Al_0.3_-SCR	5.9	64.08	12.3	17.19	0.54	---
Cu/Ti_0.6_Al_0.4_	5.33	63.82	16.83	13.03	0.41	0.57
Cu/Ti_0.6_Al_0.4_-SCR	3.18	65.45	16.6	14.27	0.51	---

* The presence of chloride is caused by its trace concentration in titanium isopropoxide used in the synthesis of aluminum doped titania precursors.

**Table 3:** XPS data of fresh catalysts.

Core Excitation	Cu/Ti		Cu/Ti_0.9_Al_0.1_		Cu/Ti_0.8_Al_0.2_		Cu/Ti_0.7_Al_0.3_		Cu/Ti_0.6_Al_0.4_	
	BE(eV) Area (%)		BE(eV) Area (%)		BE(eV) Area (%)		BE(eV) Area (%)		BE(eV) Area (%)	
Cu 2p_3/2_	930.3 932.7 934.1	2.0 91.2 6.8	930.7 932.6 935.0	2.0 90.5 7.5	930.3 932.5 935.0	4.5 93.3 2.2	930.1 932.6 935.6	4.1 92.9 3.0	930.8 932.8 935.8	5.4 91.1 2.5
Ti 2p_3/2_	458.9	100	458.5	100	458.6	100	458.6	100	458.7	100
Al 2p_3/2_	---		74.0	100	74.0	100	74.0	100	74.2	100

**Table 4:** XPS data of used catalysts.

Core Excitation	Cu/Ti-SCR		Cu/Ti_0.9_Al_0.1_-SCR		Cu/Ti_0.8_Al_0.2_-SCR		Cu/Ti_0.7_Al_0.3_-SCR		Cu/Ti_0.6_Al_0.4_-SCR	
	BE(eV) Area (%)		BE(eV) Area (%)		BE(eV) Area (%)		BE(eV) Area (%)		BE(eV) Area (%)	
Cu 2p_3/2_	930.2 932.4 935.3	3.4 95.3 1.3	930.6 933.0 935.9	4.4 94.1 1.5	931.0 933.0 935.2	2.8 92.3 4.9	931.1 932.9 935.3	6.1 92.8 1.1	931.4 932.9 935.6	8.3 87.0 4.7
Ti 2p_3/2_	459.1	100	458.5	100	459.4	100	458.9	100	458.8	100
Al 2p_3/2_	---		74.2	100	74.4	100	74.2	100	74.3	100

**Table 5:** Spectral fitting parameters for Cu 2p_3/2_ lines of fresh catalysts: binding energy (eV) and percentage of total area (in parentheses). The kinetic energy of CuLMM and Auger parameter are also listed.

Sample	Cu^0^	Cu^+^	Cu^2+^	KE of Cu L_3_M_45_M_45_	Auger Parameter
Cu/Ti	930.3	932.7	934.1	916.3	1849
	(2)	(91.2)	(6.8)		
Cu/Ti_0.9_Al_0.1_	930.8	932.6	935	916.5	1849.1
	(7.5)	(90.5)	(2)		
Cu/Ti_0.8_Al_0.2_	930.3	932.5	935	916.6	1849.1
	(4.5)	(93.3)	(2.2)		
Cu/Ti_0.7_Al_0.3_	930.1	932.6	935.6	916.3	1848.9
	(4.1)	(92.9)	(3)		
Cu/Ti_0.6_Al_0.4_	931.1	933	936	no data *	no data
	(5.4)	(92.1)	(2.5)		

* The Cu L_3_M_45_M_45_ spectra of Cu/Ti_0.6_Al_0.4_ and Cu/Ti_0.6_Al_0.4_-SCR were also measured but failed to provide useful information due to the interference with Ti 2s XPS and strong surface plasmon signals.

**Table 6:** Spectral fitting parameters for Cu 2p_3/2_ lines of used catalysts: binding energy (eV) and percentage of total area (in parentheses). The kinetic energy of CuLMM (eV) and Auger parameter (eV) are also listed.

Sample	Cu^0^	Cu^+^	Cu^2+^	KE of CuLMM	Auger Parameter
Cu/Ti-SCR	930.2	932.4	935.3	915.9	1848.7
	(3.4)	(95.3)	(1.3)		
Cu/Ti_0.9_Al_0.1_-SCR	930.6	933	935.9	916.4	1849.4
	(4.4)	(94.1)	(1.5)		
Cu/Ti_0.8_Al_0.2_-SCR	931	933	935.2	916.3	1849.3
	(4.9)	(93.3)	(2.8)		
Cu/Ti_0.7_Al_0.3_-SCR	931.4	932.9	935.3	916.1	1849
	(7.8)	(90.8)	(1.4)		
Cu/Ti_0.6_Al_0.4_-SCR	931.4	932.9	935.6	no data *	no data
	(8.3)	(87)	(4.7)		

* The Cu L_3_M_45_M_45_ spectra of Cu/Ti_0.6_Al_0.4_ and Cu/Ti_0.6_Al_0.4_-SCR were also measured but failed to provide useful information due to the interference with Ti 2s XPS and strong surface plasmon signals.

## Data Availability

The data presented in this study are available on request from the corresponding author.

## Supplementary Materials

The following are available online at https://www.mdpi.com/article/10.3390/ma14206021/s1, Table SA1: XPS O 1s data of fresh catalysts, Table SA2: XPS O 1s data of used catalysts Table SB1. XPS C 1s data of fresh catalysts., Table SB2. XPS C 1s data of used catalysts. Figure S1. TG, DTG and DSC curves for titania (A) and Ti_0.9_Al_0.1_ (B) precursors., Figure S2. C 1s XPS spectra of fresh (a) and used (b) catalysts.
