# Supplementary material for: Aluminum Doped Titania as a Support of Copper Catalysts for SCR of Nitrogen Oxides
Source: Materials (Basel). 2021 Oct 13;14(20):6021. doi: 10.3390/ma14206021 (PMC8537682; doi:10.3390/ma14206021)
Supplement: Supplementary file 1 [file materials-14-06021-s001.zip › materials-1396433-Supplementary Information-LAYOUT.pdf]

materials

MDPI

Supplementary Materials

Aluminum Doped Titania as a Support of Copper Catalysts for SCR of Nitrogen Oxides

Wojciech Guziewicz [1], Anna Białas [1,*], Bogna D. Napruszewska [2], Małgorzata Zimowska [2] and Jacek Gurgul [2]

1 Faculty of Energy and Fuels, AGH University of Science and Technology, Mickiewicza 30, 30059 Kraków, Poland
2 Jerzy Haber Institute of Catalysis and Surface Chemistry, Polish Academy of Sciences, Niezapominajek 8, 30239 Kraków, Poland
* Correspondence: anbialas@agh.edu.pl

Table SA1. XPS O 1s data of fresh catalysts.

Sample	Cu-Ti-O	oxygen in titania	defective CuO or Cu_2O	OH groups and organic contaminants
Cu/Ti	528.4	530.1	530.7	532.0
	(2.0)	(75.0)	(16.0)	(7.0)
$Cu/Ti_{0.9}Al_{0.1}$	528.2	529.7	530.4	531.9
	(3.2)	(68.0)	(18.9)	(9.9)
$Cu/Ti_{0.8}Al_{0.2}$	528.1	529.8	531.2	532.3
	(3.1)	(75.0)	(17.2)	(4.7)
$Cu/Ti_{0.7}Al_{0.3}$	528.0	529.9	530.8	532.2
	(2.6)	(58.5)	(24.4)	(14.5)
$Cu/Ti_{0.6}Al_{0.4}$	528.1	530.1	531.1	532.5
	(1.3)	(48.3)	(31.1)	(19.3)

Table SA2. XPS O 1s data of used catalysts.

Sample	Cu-Ti-O	oxygen in titania	defective CuO or Cu_2O	OH groups and organic contaminants
Cu/Ti-SCR	528.7	530.3	531.2	532.5
	(2.1)	(79.7)	(12.2)	(6.0)
$Cu/Ti_{0.9}Al_{0.1}$-SCR	528.3	530.2	531.1	532.4
	(0.9)	(68.3)	(20.4)	(20.4)
$Cu/Ti_{0.8}Al_{0.2}$-SCR	529.1	530.8	531.4	533.1
	(1.7)	(60.4)	(26.6)	(11.3)
$Cu/Ti_{0.7}Al_{0.3}$-SCR	528.6	530.4	531.4	532.8
	(2.0)	(61.3)	(27.0)	(9.7)
$Cu/Ti_{0.6}Al_{0.4}$-SCR	528.2	530.1	531.4	532.8
	(2.6)	(57.1)	(33.7)	(6.6)

Table SB1. XPS C 1s data of fresh catalysts.

Sample	C-H	C-O	O-C=O
Cu/Ti	285.0 (68.3)	286.5 (14.4)	289.0 (17.3)
Cu/$Ti_{0.9}Al_{0.1}$	285.0 (58.2)	286.3 (16.8)	289.1 (25.0)
Cu/$Ti_{0.8}Al_{0.2}$	285.0 (63.3)	286.6 (8.7)	289.1 (28.0)
Cu/$Ti_{0.7}Al_{0.3}$	285.0 (57.3)	286.4 (19.6)	289.2 (23.1)
Cu/$Ti_{0.6}Al_{0.4}$	285.0 (49.6)	286.3 (25.9)	289.5 (24.5)

Table SB2. XPS C 1s data of used catalysts.

Sample	C-H	C-O	O-C=O
Cu/Ti-SCR	285.0 (63.5)	286.3 (20.2)	289.0 (16.3)
Cu/$Ti_{0.9}Al_{0.1}$-SCR	285.0 (59.3)	286.2 (20.9)	289.3 (19.8)
Cu/$Ti_{0.8}Al_{0.2}$-SCR	285.0 (40.5)	286.1 (34.8)	289.4 (24.7)
Cu/$Ti_{0.7}Al_{0.3}$-SCR	285.0 (49.3)	286.3 (29.0)	289.5 (21.7)
Cu/$Ti_{0.6}Al_{0.4}$-SCR	285.0 (49.4)	286.2 (24.4)	289.6 (26.2)

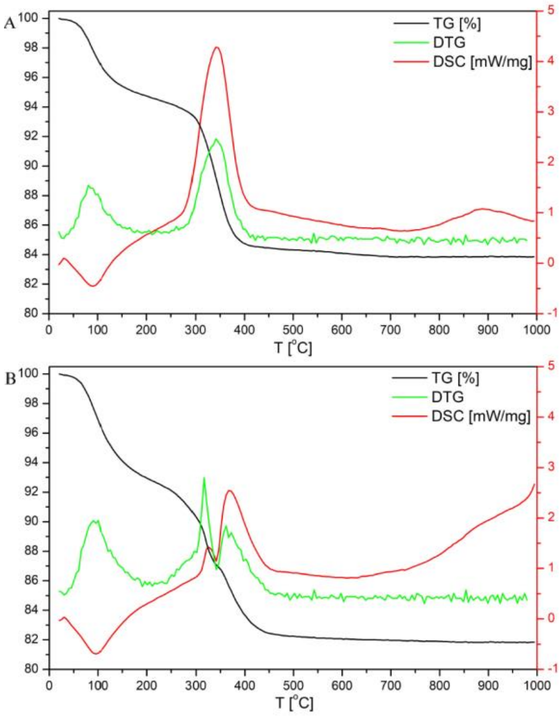

Figure S1. TG, DTG and DSC curves for titania (A) and $Ti_{0.9}Al_{0.1}$ (B) precursors.

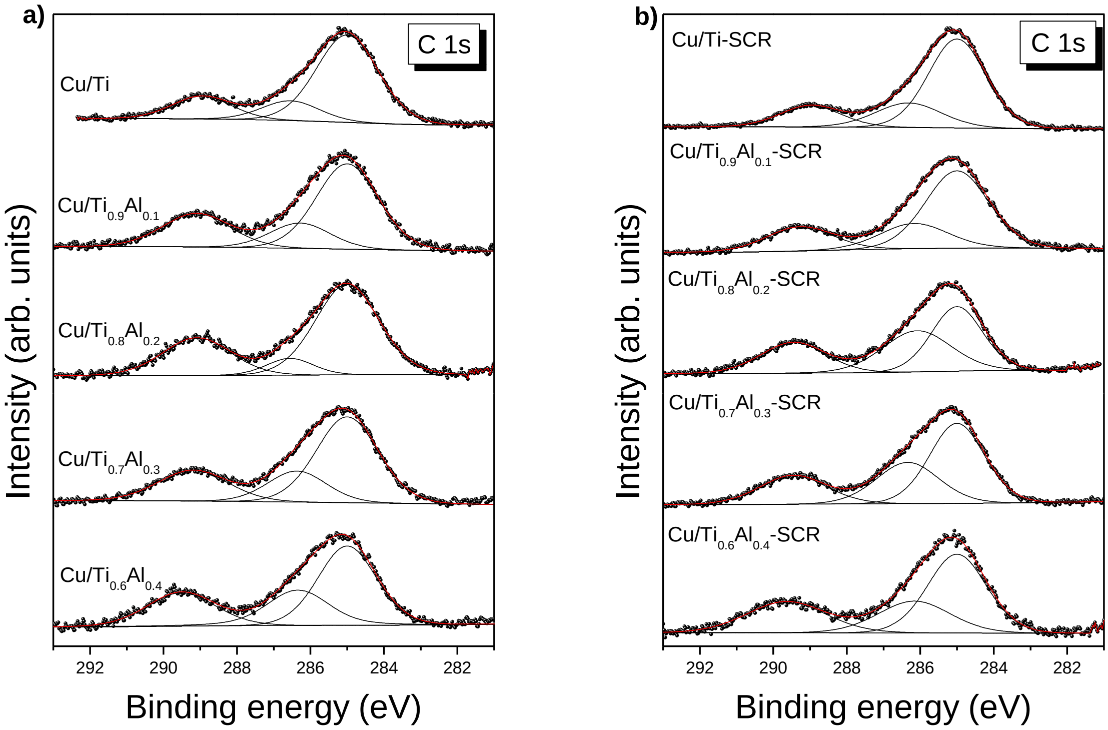

Figure S2. C 1s XPS spectra of fresh (a) and used (b) catalysts.
